# Treatment of Malarial Diseases

**Published:** 1868-12

**Authors:** 


TREATMENT OF MALARIAL DISEASES.—Dr. Sinks, in the report to the Kansas State Medical Society, says, "that notwithstanding the favorable reports of the use of the sulphites in malarious diseases, and the plausible promises that they theoretically convey, in the hands of the committee 'these salts have proved of but little value.'

"Chloroform exerts a happy influence in the cold stage of intermittents by quieting the rigors and modifying the subsequent fever, and in periodical neuralgias is quite efficient in allaying pain, but is powerless in preventing the return of the paroxysm.

"Cases occasionally presented which proved rebellious to ordinary measures, and the paroxysms continued to recur in spite of any one of the anti-periodics, however heroically administered. In these cases a combination of quinine, strychnia and arsenic rarely failed to accomplish the desired result.

"A free use of diuretics and diaphoretics in the early stage of remittent fevers, has been attended with better results than when anti-periodics were resorted to before a distinct intermission occurred. This is especially true when the eliminative organs are defective in function."—*Leavenworth Medical Herald.*

